# The Story behind the Science: The discovery of *Helicobacter pylori* CagA, the first tumorigenic bacterial protein

**DOI:** 10.1128/mbio.03641-24

**Published:** 2025-08-05

**Authors:** Rino Rappuoli, Fabio Bagnoli, Martin J. Blaser

**Affiliations:** 1 Fondazione Biotecnopolo di Siena, Siena, Italy; 2 GSK, Siena, Italy; 3 Center for Advanced Biotechnology and Medicine, Rutgers University 14863, Piscataway, New Jersey, USA; Johns Hopkins Bloomberg School of Public Health, Baltimore, Maryland, USA

**Keywords:** CagA, *Helicobacter pylori*, gastric cancer, tumorigenic bacterial protein

## Abstract

In this article, we reconstruct the history of the science that led to the discovery of CagA, the protein that causes gastric cancer, where teams in the United States and Italy made independent observations that ultimately merged. We also honor the memory of Antonello Covacci, who played an important role in this discovery and passed away prematurely in 2023.

## COMMENTARY

After the discovery of *Helicobacter pylori* in 1983 and its association with the development of peptic ulcer disease, an essential question arose. Of all of the people carrying this organism, why was it that most had no symptoms and only a relatively small percentage developed ulcers? It was an active area of investigation. Some scientists focused on differences among hosts, but several groups asked whether there were important differences among the bacterial strains. That path led two teams—one in the United States and one in Italy—to *cagA*, a gene in *H. pylori* that was the key to a novel interactive pathway with host cells. Here, we recount the early steps in the investigative process and relate some of the unexpected discoveries that ensued.

## INITIAL STUDIES

In 1988, a group in Freiburg, Germany, identified a 120 kDa protein present in 52 (83%) of the 63 *Campylobacter pylori* strains (as *Helicobacter pylori* was then called) they studied (1). The protein band was recognized by Western blotting with serum antibodies from the individual patients, but not by the 11 patients whose strain did not have the band. They deduced that the protein was on the bacterial cell surface, but there was no linkage with disease, nor could they explain why it was present on some, but not on all strains, and this observation was not advanced for several years. Also, that year, RobertLeunk and colleagues (2) reported that some, but not all, *C. pylori* strains produced a “toxin” that induced vacuolization in cultured epithelial cells.

Timothy Cover, a physician and post-doctoral fellow in the lab of Martin Blaser at the University of Colorado, was studying toxins elaborated by *Campylobacter jejuni* (3), and Blaser suggested that he look into the *C. pylori* toxin reported by Leunk and colleagues. Despite skepticism in the community about whether a toxin actually existed, Cover immediately confirmed Leunk’s finding (4). Cover’s dedicated work eventually led to the purification of the toxin molecule, which the team called VacA (5). Work conducted in parallel by Cover and Telford at Chiron Corporation in Siena, Italy, in collaboration with Montecucco and de Bernard at the University of Padua led to the cloning of the cytotoxin gene (*vacA*) and provided seminal evidence supporting the use of the antigen in a vaccine against *H. pylori* (6–8). Cover has studied VacA/*vacA* for 35 years, characterizing its structure, cellular interactions, and its biological and clinical significance (9–15). In parallel, Telford continued characterizing the toxin for many years, showing its processing, interaction with the host cell, its three-dimensional structure, and shedding light on its role in pathogenesis and as a potential vaccine antigen (16–18).

However, back in the late 1980s, having been convinced that there was vacuolating activity that differed among the (by then renamed) *H. pylori* strains, the team sought to understand its clinical significance and eventually to characterize its activity. It turned out that the questions were answered in two complementary ways.

### Thread 1: letting the patients do the work

When visiting the Centers for Disease Control (CDC) in the mid-1980s and seeking ideas about how to approach understanding bacterial (*Campylobacter*) pathogenesis, Blaser met with Dr. Charles Shepard, a leading CDC bacteriologist. Dr. Shepard had been the first to cultivate *Mycobacterium leprae* in mice (19), and in 1976, when a CDC team was trying to understand a new disease affecting American Legionnaires at their Philadelphia convention, they gave patient samples to Shepard (and his colleague Joseph McDade) to search for the etiologic agent. They put the clinical materials into embryonated eggs, and after incubation, bacteria grew out—they discovered *Legionella pneumophila* (20). When Blaser asked Dr. Shepard how to approach the problem of discovering the pathogenic mechanisms in a new pathogen, he simply said, “Let the patients do the work” (i.e., with samples from infected people, use their own immune responses to try to identify the characteristics of the agent).

A few years later, when trying to understand the vacuolating toxin, the team followed Shepard’s advice. Working with collaborators, they assembled a small library of *H. pylori* strains whose culture supernatants induced vacuolation (or not) in cultured epithelial cells. Importantly, they also had serum specimens from these same patients and others (21). Cover ran the culture supernatants on SDS-PAGE gels, electrotransferred the proteins to nitrocellulose paper, and incubated strips of the paper with serum from patients whose strain induced vacuolation or not. He found that serum IgG from patients carrying the vacuolating strains recognized a protein in the supernatants that migrated at 128 kDa that was not recognized by serum from patients whose strains were not vacuolating. In contrast, there was no consistent protein band in supernatants from non-vacuolating strains that was recognized by any serum. Those experiments showed that the 128 kDa band was strongly associated with vacuolation (4). At the time, the team thought that the 128 kDa band was the toxin, but they later showed that was not correct (22).

The most remarkable part of the study was when Cover used a tox+ supernatant (containing the 128 kDa protein) as the antigen in Western blot studies and examined the serum responses of 166 persons who had defined gastric histopathology. Among 49 people who were not carrying *H. pylori* (controls), only 3 (6%) recognized the protein, but among 74 people who had *H. pylori* and had the histological signature (gastritis alone) of carrying the organism, 45 (61%) recognized the protein. Importantly, of 31 patients who had a duodenal ulcer, all (100%) recognized the protein, and of 12 with gastric ulcer, 10 (83%) also recognized the band. In summary, they found that serum IgG recognition of the 128 kDa protein was strongly associated with ulcer disease, especially duodenal ulcer. On 9 August 1989, they submitted their paper describing these findings to *Infection and Immunity,* and it was published the following year (4).

### Thread 2: molecular cloning

**Fig 1:**
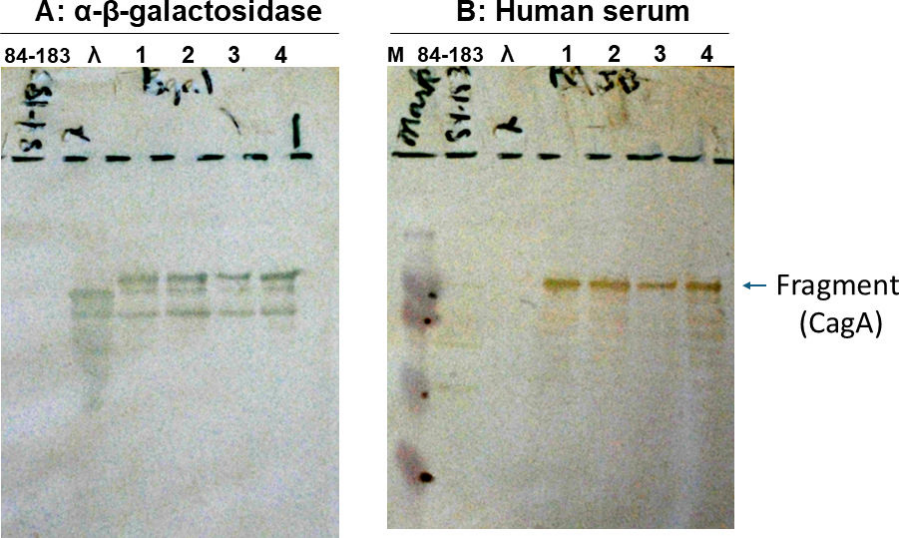
Gel of the initial cloned cagA product in λgt11 on 20 July 1989. A library of *H. pylori* strain 84-183 sonicated DNA fragments was cloned into *E. coli* using phage λgt11, and expressing plaques from the library were screened by plaque blot using serum from an *H. pylori*-seropositive person (MJB). Clone 7/2-1 was isolated and used as an antigen in Western blot analysis. The blots were screened with serum from a rabbit immunized against *E. coli* β-galactosidase (**A**) or with human serum (**B**). A lysate of strain 84-183 and an empty λgt11 clone were used as controls (first two lanes from left in panel A and lanes 2 and 3 in panel B). Panel A shows the β-galactosidase fusion protein, and panel B shows that it was recognized by human serum. The 0.6 kb insert from a colony recognized by the serum was then cloned into pUC19 to create pYB1, and the remaining cagA gene was identified by Southern hybridization of 84-183 λZapII clone pMC3 with pYB1, as described (24).

In 1988, Blaser had returned from a sabbatical at Rockefeller University, where he worked with Professor Emil Gotschlich. Under his tutelage, in another *Campylobacter* species (*Campylobacter fetus*), Blaser cloned an important gene, which they called *sapA* (23). They accomplished this by creating an expression library of *C. fetus* genes in *Escherichia coli*, using the phage λgt11, and screened the library with rabbit antiserum that had been raised against the SapA protein. Based on this success, Blaser decided to make an expression library of *H. pylori* genes in λgt11 as well, and the team then screened this library using serum from a person who was positive for *H. pylori*. That person was Blaser, because earlier, with GuillermoPerez-Perez, the team had created a serological test to detect antibodies to *H. pylori* (21), and Blaser’s serum was strongly positive. When the library was screened in July 1989, the team identified a colony that expressed a recombinant protein strongly recognized by Blaser’s serum (Fig. 1); later, they found that that protein was a large fragment of the *cagA* gene product (see below).

## ADVANCES IN EUROPE

On 10 August 1991, 2 years after the U.S. team submitted their findings to *Infection and Immunity*, a paper was published in *The Lancet* by Crabtree and colleagues in Leeds and Bradford, England. The team studied IgA production by gastric antral biopsies in 100 patients undergoing upper GI endoscopy who were found to have gastritis alone or duodenal ulcer (25). Crabtree’s team found that the antral biopsies contained antibodies that recognized a number of *H. pylori* proteins that did not differentiate the patients with gastritis alone from those with duodenal ulcer. However, they found recognition of a protein that migrated at about 120 kDa in 63% of the 51 gastritis patients and 100% of the 25 patients with duodenal ulcers; this was the most remarkable finding! The U.S. scientists immediately deduced that this was the same protein as their 128 kDa protein, and the percentages of recognition were virtually identical to the ones found in the U.S. subjects. That two separate research teams studying host antibody responses to *H. pylori*, either IgG in the blood or IgA in gastric secretions, found nearly identical proportions of responses in gastritis and ulcer patients meant that they had independently identified a reproducible finding of medical significance. From that point on, we knew that we were on the right track!

In 1988, scientists in Siena were also interested in the newly discovered (2) cytotoxic activity of *C. pylori* (26). Studying samples from patients undergoing upper gastroduodenal endoscopy, Figura and colleagues examined cytotoxic activity by culture supernatants. In strains isolated from 24 patients with duodenal ulcers, 16 (67%) had cytotoxic activity versus 16 (30%) in 53 patients with gastritis only, suggesting that the toxic substance could have a role in the development of peptic ulcers. Although no molecule was detected, this was a first step linking variation in *H. pylori* strains and ulcer disease. Crabtree and colleagues, later working with Figura, continued to examine the relationship of their 120 kDa protein with ulcer disease (27). Also, a German group continuing the work of Apel et al. (1) found sero-reactivity of the 120 kDa protein in >90% of infected subjects but made no association with specific clinical conditions (28).

## CLONING OF *cagA* IN NASHVILLE

After 1989 (and moving to Vanderbilt University), the work in the two threads in the Blaser lab combined. They began to sequence the gene in the strongly reacting λgt11 clone and were making slow progress, but when Tummuru, a new post-doctoral fellow, entered the lab in 1990, the project advanced quickly. He created a second library using λZapII and also screened that with Blaser’s serum. By analyzing clones that expressed antigenic proteins, the team was able to piece together the sequence of the full-length gene, which encoded a protein of ~131 kDa, very similar to the 128 kDa band identified by Tummuru et al. (24). Importantly, when they studied 32 *H*. *pylori* strains using colony hybridization with the recombinant clone, 19 hybridized; all 19 possessed the 128 kDa protein, in comparison to none of the non-hybridizing strains. These clear-cut results confirmed that the team had cloned the gene encoding the 128 kDa protein identified in the 1990 paper (4). At the time, they recognized that the protein had three EPIYA-repeated motifs of unknown function, which were later found to have great significance as tyrosine-phosphorylation domains.

## CLONING OF *cagA* IN SIENA

In the early 1990s, the research team at Chiron Corporation in Siena, under the leadership of Rappuoli, was collaborating with Figura, trying to understand which bacterial factors were involved in *Helicobacter pylori* virulence and tumorigenesis, with the ultimate goal of making a vaccine to prevent cancer. Indeed, although antibiotic therapy is relatively effective at eliminating the organism, *H. pylori* infection usually remains asymptomatic for multiple decades, and cancerous or pre-cancerous lesions can occur during the long exposure to the organism. Quite soon, Covacci identified, cloned, and sequenced a 128 kDa immunodominant antigen, which was present mostly in isolates retrieved from duodenal ulcer patients. The protein was shown to be associated with the secretion of IL-8 from gastric epithelial cells, promoting neutrophil chemotaxis and activation. Before the publication in *PNAS* in 1993 (29), the Siena team became aware that in the United States, Tummuru had also completed the sequencing of the gene encoding the 128 kDa protein and had tentatively called it “tagA” (toxin-associated gene A). After their manuscript was submitted for publication to *Infection and Immunity* in December 1992 (24), the U.S. team also became aware that the group in Siena had identified the same gene in *H. pylori* and also found its association with cytotoxin expression (29). The Italian team had named the gene “*caiA”* (cytotoxin-associated immunodominant gene A). Together, the two teams believed that the field would move forward better with a common name, so they agreed on the new name *cagA*, which used three letters from each of the tentative names (24, 29), and *cagA* has been the undisputed name ever since.

## NEXT STEPS IN THE UNITED STATES

In 1995, the U.S. team published a number of papers that began to fill in the details of the clinical and pathogenetic significance of *cagA*. Cover led a team to use a recombinant *cagA* fragment to encode an antigen to detect serum IgG antibodies to CagA in an ELISA (30); those studies confirmed the strong association of *cagA*+ strains and peptic ulcer disease that had been detected by the Western blotting studies in 1989 (4). Working with Harry Kleanthous at Oravax, the U.S. team used a longer cloned *cagA* fragment as the basis for a serological test to determine which people had antibodies to CagA and thus were carrying a *cagA+* strain (31). They showed that the test was both sensitive and specific, using sera from patients with known *cagA*+ strains and longitudinal specimens, which also demonstrated that seropositivity was stable for >7 years. In studies involving sera from 103 Japanese-American men who developed gastric cancer and their matched controls from a longitudinal cohort (26), adding cagA seropositivity to *H. pylori* status essentially doubled the risk for gastric cancer, especially for the most common intestinal type of adenocarcinoma (OR 2.3 [1.0–5.2]). Thus, in addition to the originally determined increased risk for ulcer disease associated with carrying a *cagA*+ strain, they showed that there also was increased risk for the most common type of gastric cancer; this finding was confirmed 2 years later by Parsonnet and colleagues at Stanford (32). In addition to finding the relationship of *cagA*+ strains with gastric cancer, the U.S. team showed relationships with heightened gastric inflammation (33), production of the pro-inflammatory cytokine IL-8 (34), and the development of atrophic gastritis, a key step in the pathway toward gastric cancer (35)

Based on the continuing evidence that *cagA* had medical significance, Tummuru sought to characterize its adjacent genes. By chromosome walking, he had cloned a 4.5 kb region of the *H. pylori* genome that hybridized with a probe upstream of *cagA*. Sequencing this fragment revealed two open reading frames in the opposite orientation from *cagA*, which the team called *picA* and *picB (*see below). Although the *picA* product had no homology to known proteins, the predicted *picB* product had strong homology to *Bordetella pertussis* toxin secretion protein PtlC and with *Agrobacterium tumefaciens* virB4, which is part of a type IV secretion system (T4SS); this was the first evidence that *H. pylori* had a homolog to a type IV secretion system gene (36). In 55 *H*. *pylori* strains tested, the presence of *cagA* and *picB* was perfectly correlated. Importantly, the team created a *picB* mutant and examined its interactions with gastric epithelial cells in culture; while the wild-type strain induced production of the pro-inflammatory cytokine IL-8, this was lost in the *picB* mutant. That was the basis for calling the gene *picB* (permits induction of cytokine). The gene is now known as *cagE*, and it was later shown to be required for type IV secretion system activity (see below), transporting the CagA protein into epithelial cells.

## UNDERSTANDING THE FUNCTION OF THE TYPE IV SECRETION SYSTEM IN SIENA AND ELSEWHERE

Later, the Siena and Nashville teams, with colleagues from Stanford, collaborated on a study to better characterize *H. pylori* virulence mechanisms in experimentally inoculated mice (37). Among other findings was the observation that a strain in which *cagA* was knocked out showed the same level of epithelial cell damage and inflammation as the wild-type strain. This confirmed that the presence of *cagA* was not essential for pathogenesis. However, Covacci decided to focus on CagA, and in collaboration with the laboratory of Falkow at Stanford, was able to link the protein to cancer and identify many of the mechanisms by which CagA favors tumorigenesis (38). Initially, they demonstrated that the *cagA* gene is contained in a pathogenicity island encoding 31 genes (called the *cag* pathogenicity island, *cag*PAI)*,* including those for a type IV secretion system (39). In St. Louis, the team of Douglas Berg also defined the cag pathogenicity island (40). Between the years 1996 and 2002, work conducted mainly at Stanford and Siena (38, 41, 42) and confirmed by others (43–45) demonstrated that upon attachment of *H. pylori* to gastric epithelial cells, CagA gets phosphorylated, and this event was linked to induction of signal transduction and dramatic morphological changes of the host cell, which was named the “hummingbird” phenotype (42). Translocation of CagA to the host cells was then found to occur by means of the type IV secretion system, which forms a needle that injects the bacterial protein into the host cells (44, 45). Markus Stein and Fabio Bagnoli in Covacci’s team discovered that CagA is phosphorylated by the host Src-family kinase at tyrosine residues in the repeated EPIYA motifs and that this event was necessary to induce the hummingbird phenotype (38, 40).

## BROAD ADVANCES IN NASHVILLE

An important and initially unexpected advance came when Peek and Blaser began to explore whether *cagA*+ strains were associated with any of the increasingly prevalent conditions affecting the esophagus: reflux esophagitis (also called GERD), Barrett’s esophagus, and adenocarcinomas arising in the proximal stomach or distal esophagus (called GE junction adenocarcinomas). Working with colleagues at the Cleveland Clinic, the National Cancer Institute, and in the Netherlands and Japan, the team found that the presence of gastric colonizing *cagA+ H. pylori* strains, as detected by the presence of serum antibodies to the CagA protein, was inversely associated with each of these conditions (46–50). Although by their nature, these studies could not establish a causal (protective) role of *cagA+ H. pylori* strains, the consistency of the findings and multiple later independent confirmations of the phenomenon (51–53) began to position *cagA*+ strains in a new light. In addition to the disease costs that were associated with gastric colonization by these organisms in terms of adenocarcinomas of the distal stomach and peptic ulcer disease (all involving accelerated gastric pathology) (54, 55), there were strong protective associations with the nested diseases of the esophagus. *H. pylori* strains lacking *cagA* had little impact on any of these disease conditions, for reasons that soon became obvious (see “Linking CagA and gastric cancer at the molecular level at Stanford and in Siena”).

## LINKING CagA AND GASTRIC CANCER AT THE MOLECULAR LEVEL AT STANFORD AND IN SIENA

**Fig 2:**
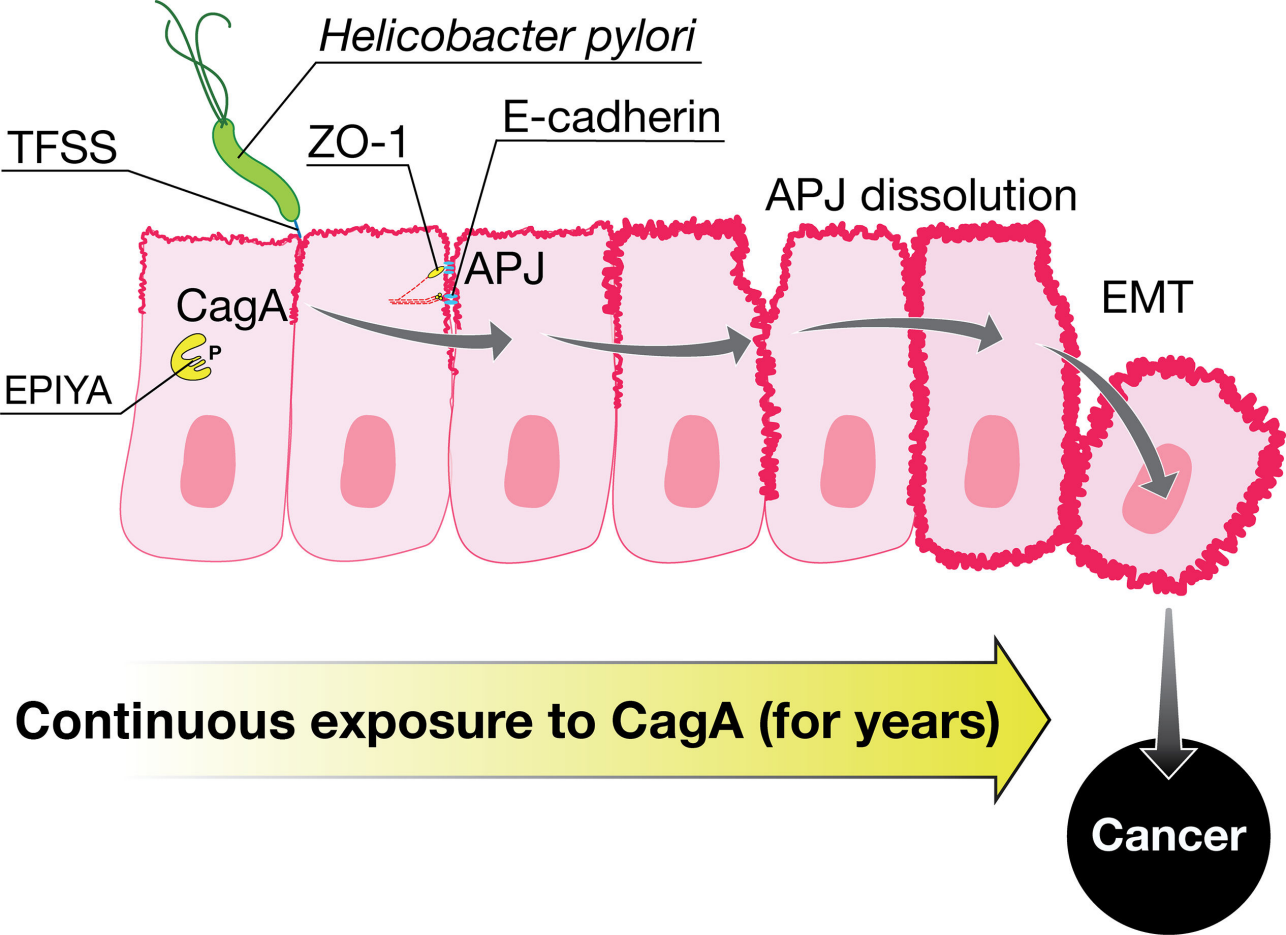
Prolonged exposure to CagA may induce cancer. CagA is injected by *H. pylori* through a type IV secretion system into gastric epithelial cells. CagA intracellular activity causes apical junction (APJ) dissolution and epithelial-to-mesenchymal transition (EMT).

Using polarized MDCK cells, the team led by Amieva and Falkow at Stanford, in collaboration with Covacci, showed that CagA colocalizes and interacts with the zonula occludens protein ZO-1 and recruits polarity-associated serine/threonine kinase MARK2 from the cytoplasm to the membrane near the bacteria. These interactions cause apical junction dissolution and inhibition of tubulogenesis as well as cellular dedifferentiation (56, 57). Bagnoli, during his postdoctoral work in Amieva’s lab, transfected the *cagA* gene into MDCK cells and showed that the protein is sufficient to induce loss of apicobasal polarity and cell–cell adhesion. Cells expressing CagA extended migratory pseudopodia, degraded basement membranes, and acquired an invasive phenotype. Bagnoli and Amieva proposed for the first time that all these attributes resemble epithelial to mesenchymal transition, a process at the basis of cellular transformation and metastasis ([53] and Fig. 2), later confirmed by others (58, 59). CagA was also shown to localize at the cellular junctions by means of its N-terminal region, while the C-terminus, containing the EPIYA motifs, interferes with intracellular signaling and causes its biological effects (60). Expression of the CagA C-terminal domain induces pseudopodial activity but is not sufficient to induce cell migration. Neither domain is sufficient to induce these phenomena, but when co-expressed in *trans*, the function of the full-length protein becomes re-established (60). Finally, CagA was found to be able to bind and interfere with the function of the protein ASPP2. The latter protein is involved in the suppression of the apoptosis-stimulating p53 protein. After having bound CagA, ASPP2 recruits p53 and inhibits its apoptotic function. Furthermore, CagA diminishes p53 activity by increasing its degradation (61). Derailment of the p53 tumor suppressor resembles DNA tumor viruses, another finding linking CagA with the increased risk of gastric cancer.

## RESEARCH AND DEVELOPMENT OF NEW THERAPEUTIC APPROACHES AGAINST *H. pylori*

The U.S. team filed patent applications related to their discoveries about cagA and vacA; eventually, seven U.S. patents were issued; as part of the collaborations, the Siena group at Biocine (later to become part of Novartis) licensed several of these patents from the U.S. group (Vanderbilt University) for the purpose of creating vaccines. Although their work was thus intertwined, the research paths diverged; in Siena, the focus was on molecular biology, pathogenesis, and development of a therapeutic small molecule and a vaccine.

The Siena group then collaborated with the groups led by Del Giudice at Novartis and Malfertheiner at Ludwig-Maximilians-Universität in Munich to develop a vaccine containing CagA, the vacuolating cytotoxin A (VacA), and neutrophil-activating protein formulated with aluminum hydroxide (62). A phase 1 trial in 57 *H*. *pylori*-negative healthy volunteers tested two dosages (10 and 25 mcg) of each antigen using three schedules (0, 1, and 4 months; 0, 1, and 2 months; and 0, 1, and 2 weeks) versus alum alone. The vaccine was safe and induced an antigen-specific antibody response in 86% of subjects and a cellular response (62). The vaccine was subsequently tested in a phase 1-2 trial in *H. pylori*-negative adults who were 18–40 years old; they received three intramuscular doses of either placebo or vaccine according to the 0-, 1-, and 2-month schedule (63). Of the 63 subjects included in the study, 27 received a placebo and 36 received the vaccine. One month after the last immunization, 34 participants (19 vaccinated and 15 placebo) were challenged with a CagA-positive *H. pylori* strain, which induced transient mild-to-moderate epigastric symptoms in all but one subject. Three months after the challenge, 6 (32%) of 19 in the vaccinated group and 6 (40%) of 15 in the placebo group remained positive for *H. pylori*. Despite increased antibody titers, the vaccine did not show any significant protection against the challenge. Human challenge studies, although crucial for supporting the development of new vaccines and drugs, sometimes do not fully represent disease mechanisms and require time to be properly established. The low economic attractiveness of an *H. pylori* vaccine did not allow additional tests to be performed, and therefore, no vaccine against *H. pylori* has been developed.

## POST-SCRIPT: LATER WORK ON CagA

In the United States, the major focus was on the clinical significance of *cagA*+ strains and the biology of their interaction with the host. Having found an inverse association between *cagA*+ strains and esophageal disease, Blaser and colleagues (now at New York University) next studied whether there might be a similar relationship with asthma, because of the well-known association of GERD with asthma. They indeed found an inverse association of the carriage of *cagA*+ strains and asthma (64, 65), as well as allergic disorders (64). These epidemiologic studies were followed by brilliant studies of *H. pylori* infection in experimentally induced asthma in mice that were led by Anne Mueller in Zurich. Although the role of *cagA per se* was not determined, the experimental studies provided consistent evidence that *H. pylori* colonization protected against asthma (66) and identified a mechanism through the involvement of dendritic cells in programming the differentiation of T reg cells (67). Since other epidemiologic studies showed that *cagA*+ strains were rapidly disappearing from human populations (68), we may be losing an important defense against asthma and allergic diseases.

Other work has highlighted how *cagA+ H. pylori* strains interact with their human hosts. Aras and colleagues (69), studying a component of the type IV Cag secretion system (CagY, a VirB10 homolog), provided evidence for the antigenic variation of this host-exposed bacterial structure. Further work dissected the differential interaction of *cagA*+ strains with the expression of epithelial cell protein cyclin D, critical in cell cycle dynamics (70), activation of the ERK-phosphoprotein (71), and involvement of JAK/STAT signaling based on the status of the tyrosine phosphorylation domains (72). More than 25 years after the discovery of *cagA*, studies showed that the molecular structure of the type B tyrosine phosphorylation domain determines its host-interacting properties, associated with clinical outcome (73). Finally, the structure and function of the T4SS, which strongly interacts with human cells, are being defined with increased resolution by Cover’s team (74–77).

### Conclusions

The discovery of *cagA* in *Helicobacter pylori* and its biological significance was a multi-year process involving several investigative teams. The work began independently, then briefly joined and diverged again. Throughout, there was a healthy and respectful competition, and we are proud of how we worked together. The discoveries of Covacci have advanced CagA as a strong candidate for a vaccine and an excellent target for drugs for the prevention and treatment of gastric cancer. The *in vitro* transformative changes of epithelial cells induced by a single protein also represent an excellent model to dissect the molecular mechanisms that eventually lead to cancer.

The initial work by the two teams has opened up a broad field touching many areas of biological science and medicine. In the 32 years since *cagA* was discovered and named, there have been 4,460 papers in which *cagA* appears in the title or abstract, with a steady 150–200 papers a year since 1999 (PubMed, 15 June 2025). There have also been 635 papers for the *cag* pathogenicity island, respectively (4,704 with either term).

This review has focused almost exclusively on the work of the two investigative groups that made the largest initial contributions, but there have been a large number of scientists who have broadly moved the field of *cagA* biology forward with their exciting discoveries. Some recent excellent reviews of the field (78–80) highlight the continuing advances in this clinically relevant model system of host-microbial interaction, and to complete this historical review, five relevant patents are cited (81–85).

Covacci, a visionary scientist, passed away prematurely on 2 February 2023 at the age of 65. His main life goal, both professionally and personally, was to support science and the scientists he deemed worthy.
